# Design and Functional Evaluation of an Embedded Multiparametric Platform for Fermentation Monitoring

**DOI:** 10.3390/s26216853

**Published:** 2026-10-29

**Authors:** Juan L. López, Lukas Espinoza-Villarroel, Ángela Contreras, Miguel Riffo-Parra, Daniel Toral Acosta, Romeo Selvas Aguilar, Ricardo Chapa Garcia, Axel Quinteros-Castillo

**Affiliations:** 1 Centro de Innovación en Ingeniería Aplicada, Universidad Católica del Maule, Av. San Miguel 3605, Talca 3460000, Chile; 2 Department of Computer Science and Industries, Universidad Católica del Maule, Av. San Miguel 3605, Talca 3460000, Chile; 3 Departamento de Energías Renovables, Tecnológico Nacional de México, Campus Progreso, Progreso 27320, Yucatán, Mexico; 4 Centro de Biotecnología de los Recursos Naturales, Escuela de Ingeniería en Biotecnología, Departamento de Ciencias Forestales, Facultad de Ciencias Agrarias y Forestales, Universidad Católica del Maule, Av. San Miguel 3605, Talca 3460000, Chile; 5 SECIHTI, Universidad Autónoma de Nuevo León, Pedro de Alba S/N, Ciudad Universitaria, San Nicolás de los Garza 66455, Nuevo León, Mexico; 6 Facultad de Ciencias Físico-Matemáticas, Universidad Autónoma de Nuevo León, Pedro de Alba S/N, Ciudad Universitaria, San Nicolás de los Garza 66455, Nuevo León, Mexico

**Keywords:** embedded systems, multiparametric monitoring, fermentation monitoring, physicochemical sensing, distributed acquisition, wine fermentation, proof-of-concept

## Abstract

The continuous monitoring of physicochemical variables can provide temporally resolved information during fermentation processes while reducing dependence on discrete manual measurements. This work presents the design, implementation, and functional proof-of-concept evaluation of an embedded multiparametric platform for continuous monitoring in laboratory-scale fermentation environments. The platform integrates sensing, embedded data acquisition, local processing, chronological data organization, and automatic storage within a distributed architecture. Temperature, pH, dissolved oxygen (DO), and oxidation-reduction potential (ORP) were monitored using commercially available sensing devices integrated into a stainless-steel fermentation reactor with an approximate nominal capacity of 25 L and a working volume of 20 L. An Arduino Uno was used for sensor interfacing and data acquisition, while a Raspberry Pi 4 performed local processing, chronological organization, and automatic storage of the acquired information. The experimental evaluation comprised preliminary operation under controlled conditions followed by a Cabernet Sauvignon wine fermentation used as a real-process operating scenario to assess the integrated functionality of the platform under enological conditions. During the reported experiments, the sensing, acquisition, communication, processing, and data-management subsystems operated jointly and generated continuous, chronologically structured multiparametric records. The monitored channels exhibited distinct temporal patterns with comparatively gradual variations in temperature and pH and shorter-duration fluctuations in ORP and DO. The experimental protocol was designed to evaluate integrated system operation rather than the metrological performance of the individual sensing channels. Consequently, the recorded values should be interpreted as proof-of-concept monitoring data and not as validated analytical determinations of enological parameters. In particular, the absolute pH values recorded during the initial stage and the short-duration variations observed in ORP and DO cannot be unambiguously interpreted without synchronized measurements against reference instrumentation. The results demonstrate the feasibility of integrating multiple physicochemical sensing channels with embedded acquisition, processing, and automatic data-management capabilities within a common experimental platform. The proposed system therefore constitutes a laboratory-scale technological proof-of-concept and provides a basis for future work involving quantitative sensor characterization, repeatability and drift assessment, and validation against reference instrumentation before analytical or industrial-scale application.

## 1. Introduction

Alcoholic fermentation is one of the central stages of winemaking because the conversion of grape sugars into ethanol and carbon dioxide occurs together with numerous biochemical transformations that influence the physicochemical and sensory characteristics of the resulting wine. The evolution of this process depends on the interaction between yeast metabolism and the surrounding fermentation environment, making the observation of physicochemical conditions an important component of experimental and production-scale enology.

Among the variables associated with fermentation evolution, temperature, pH, dissolved oxygen (DO), and oxidation–reduction potential (ORP) provide complementary information regarding thermal conditions, acid–base equilibrium, oxygen availability, and the redox state of the medium. Temperature influences microbial activity and fermentation kinetics and can affect the composition of the resulting wine [1,2]. The evolution of pH is related to several chemical and microbiological phenomena and has important implications for wine composition, microbial stability, and organic-acid metabolism [3,4,5]. Oxygen availability also influences yeast metabolism and wine properties [6,7,8], while ORP provides information about changes in the oxidative or reductive conditions developing throughout fermentation [9,10,11,12].

Monitoring these variables conventionally involves periodic sampling and laboratory analysis. Although discrete measurements remain essential for analytical characterization, they provide observations only at selected times and may therefore omit changes occurring between sampling intervals. Continuous monitoring offers a complementary approach by preserving the temporal evolution of the process and reducing the need for the repeated manual acquisition of individual measurements.

Advances in sensing technologies and embedded electronics have progressively expanded the possibilities for continuous enological monitoring. Electrochemical sensors have been investigated for different stages of wine production [13,14], while optical approaches have enabled a non-invasive monitoring of variables such as dissolved oxygen during fermentation [15]. Distributed monitoring architectures have also been proposed for wine-related applications, including wireless sensing systems for temperature and headspace monitoring [16]. In parallel, spectroscopic monitoring combined with chemometrics has been used to follow chemical changes continuously during fruit-wine fermentation [17]. More recently, real-time gas monitoring has been incorporated into instrumented wine tanks to follow oxygen and carbon-dioxide evolution during vinification [8]. These developments illustrate the transition from isolated measurements toward continuously acquired digital information.

A parallel evolution has occurred in multiparametric and multisensor approaches. The combination of different sensing principles can provide complementary information that cannot be obtained from a single measurement channel. Multiparametric analyzers based on electrochemical sensing have been investigated for process control in the wine industry [18], while multisensor strategies and data-fusion approaches have been explored for broader wine characterization [19]. More recently, continuous digital information has also provided the basis for developments involving predictive modeling and intelligent decision-support tools in viticulture and winemaking [20,21].

These developments show that the technological challenge in fermentation monitoring is not restricted to the availability of individual sensing elements. An equally important aspect is the integration of different measurement channels with data acquisition, processing, chronological organization, and storage within a common experimental infrastructure. Such integration is particularly relevant when the objective is to preserve the simultaneous temporal evolution of several physicochemical variables throughout a fermentation experiment.

Despite considerable progress in sensing and digital monitoring technologies, existing studies address a broad range of objectives, measurement principles, and implementation scales. Sensor-oriented contributions frequently emphasize the analytical characteristics of a particular measurement principle, whereas other approaches focus on communication, distributed sensing, or advanced data analysis. Consequently, there remains practical interest in laboratory-scale experimental platforms in which commercially available sensing technologies can be integrated with embedded acquisition and information-management capabilities for continuous multiparametric fermentation monitoring.

This requirement is also relevant for experimental research and small-scale winemaking environments, where flexible monitoring infrastructures may facilitate the systematic acquisition of fermentation data without requiring highly specialized instrumentation for every monitored variable. In Chile, this context has particular relevance because the Maule Region represents a major fraction of national wine production [22]. Studies of spontaneous fermentation using grape cultivars from the Maule Region further illustrate the local relevance of monitoring physicochemical and microbiological changes during fermentation [23]. Nevertheless, the present study does not attempt to demonstrate an economic advantage over commercial monitoring equipment; rather, the use of commercially available components is considered from the perspective of implementation accessibility and experimental flexibility.

The platform was motivated by the need for accessible and flexible experimental monitoring infrastructure in small-scale enological environments. Cabernet Sauvignon fermentation was selected as a representative operating scenario. Although grape sugar and nitrogen availability are relevant to fermentation performance, this study does not demonstrate adaptation to a particular regional climatic condition.

Motivated by these considerations, the objective of this work is to design, implement, and functionally evaluate an embedded multiparametric platform capable of integrating continuous sensing, data acquisition, local processing, chronological organization, and automatic data storage within a laboratory-scale fermentation environment. The proposed architecture integrates temperature, pH, dissolved oxygen (DO), and oxidation–reduction potential (ORP) sensing channels within a common embedded monitoring infrastructure.

A Cabernet Sauvignon wine fermentation was employed as a real-process operating scenario to evaluate the integrated operation of the platform under enological conditions. Accordingly, the experimental objective is to determine whether the sensing, acquisition, processing, communication, and data-management subsystems can operate jointly and continuously during a fermentation process.

The scope of this study is therefore restricted to functional proof-of-concept evaluation. It does not aim to establish metrological equivalence with reference instrumentation, validate the individual sensing channels for an analytical determination of enological parameters, or demonstrate the industrial qualification of the platform. Measurement uncertainty, systematic repeatability, long-term drift characterization, and quantitative comparison against traceable reference instrumentation remain outside the experimental scope of the present work.

Within this framework, the main contributions of the present work are outlined below:

- The design and implementation of an embedded platform that integrates multiple physicochemical sensing channels within a common fermentation monitoring infrastructure;
- The implementation of a distributed acquisition and processing architecture that separates sensor acquisition from information organization and storage;
- The generation of chronologically structured multiparametric records containing temperature, pH, ORP, and DO during a laboratory-scale wine fermentation used as a real-process operating scenario for the integrated platform;
- The functional evaluation of the complete monitoring chain under controlled and enological conditions together with an explicit analysis of its present scope and limitations.

The contribution should therefore be understood in terms of system integration and functional operation rather than the introduction of a new sensing principle. The proposed platform provides an experimental infrastructure for continuously acquiring complementary physicochemical information and establishes a basis for subsequent work involving quantitative sensor characterization, larger-scale evaluation, and, after appropriate validation, more advanced fermentation supervision strategies.

The remainder of this paper is organized as follows. Section 2 describes the methodological framework, embedded monitoring platform, distributed architecture, experimental fermentation environment, data-acquisition strategy, and functional validation procedure. Section 3 presents the experimental results and functional evaluation, including the temporal behaviour of the monitored variables and the limitations of the current implementation. Finally, Section 4 summarizes the principal findings and identifies the steps required for the subsequent development of the platform.

## 2. Materials and Methods

### 2.1. Methodological Overview

The methodological approach adopted in this study was conceived to develop and functionally evaluate an embedded multiparametric monitoring platform for the continuous observation of physicochemical variables involved in alcoholic fermentation. Rather than focusing on the isolated implementation of individual sensors, the methodology considered the monitoring system as an integrated experimental infrastructure in which sensing, embedded acquisition, information management, and fermentation experiments operate as complementary components.

The workflow comprised five consecutive stages: identification of the physicochemical variables to be monitored; design of the integrated monitoring platform; implementation of the distributed acquisition and processing architecture; integration of the platform into a laboratory-scale fermentation environment; and functional evaluation under controlled and enological conditions. The experimental objective was to verify coordinated system operation and continuous information acquisition rather than provide a metrological characterization of the individual sensors.

Figure 1 summarizes the methodological workflow used in this study.

### 2.2. Embedded Multiparametric Monitoring Platform

The experimental system consists of an embedded multiparametric monitoring platform designed for the continuous observation of temperature, pH, dissolved oxygen (DO), and oxidation–reduction potential (ORP). The platform integrates sensing devices, embedded electronics, data acquisition, local processing, and information management within a coordinated monitoring infrastructure (Figure 2).

From a functional perspective, the platform comprises four complementary subsystems: (i) a sensing subsystem responsible for measuring the selected physicochemical variables; (ii) an embedded acquisition subsystem responsible for sensor interfacing and data acquisition; (iii) a processing and information-management subsystem responsible for chronological organization and storage; and (iv) an experimental fermentation environment in which continuous monitoring is performed.

The modular organization allows the sensing and computational functions to remain separated while operating within a common monitoring workflow. The functional organization described above was implemented through the distributed embedded architecture presented in the following subsection.

The component identification reported in Table 1 was cross-checked against the technical documentation retained in the original project bibliography. The DS18B20 temperature sensor and RS-LDO-N01-1 dissolved-oxygen transmitter are documented in [24,25]. The available project record for the pH channel identifies a BNC electrode/interface kit supplied by Mechatronic Store but does not report an original equipment manufacturer or model number [26]. The corresponding model and manufacturer for the ORP sensing channel could not be established from the source records and are therefore reported as not specified rather than inferred.

The pH channel uses a PH-4502C analog conditioning module connected to the Arduino Uno analog-to-digital interface. The DS18B20 temperature sensor communicates through 1-Wire, while the Renke RS-LDO-N01-1 dissolved-oxygen transmitter communicates digitally using Modbus RTU over a USB–RS485 interface connected to the Raspberry Pi. The original pH electrode manufacturer and the ORP probe specifications could not be established from the retained records.

### 2.3. Distributed Embedded System Architecture

The proposed platform adopts a distributed embedded architecture composed of coordinated sensing, acquisition, processing, and information-management functions. The architecture is organized into two principal computational levels. The first level is dedicated to sensor interfacing and data acquisition, whereas the second level performs data processing, chronological organization, and permanent storage.

The acquisition level continuously interrogates the sensing subsystem and transfers the resulting digital information to the processing level. The processing level receives the measurements and organizes the experimental information according to the acquisition sequence. To implement this architecture, an Arduino Uno microcontroller (Arduino SA, Chiasso, Switzerland) was used for sensor interfacing and data acquisition, whereas a Raspberry Pi 4 single-board computer was used for local processing and automatic data storage.

Local data-management routines were implemented in Python version 3.X to generate structured files organized chronologically by acquisition date. This implementation supports continuous experimental logging while preserving the temporal order of the monitored variables. Once the embedded architecture had been established, the monitoring platform was integrated into the experimental fermentation environment described below (Figure 3).

### 2.4. Experimental Fermentation Environment

The monitoring platform was implemented in an instrumented laboratory-scale stainless-steel fermentation reactor with an approximate nominal capacity of 25 L. Fermentation assays were conducted in batch mode using a working volume of 20 L. The reactor provided the physical environment in which the sensing subsystem and distributed embedded architecture operated during the experiments.

The reactor configuration allowed for the simultaneous installation of the sensing devices used to monitor temperature, pH, dissolved oxygen, and ORP. The sensing elements remained integrated with the fermentation environment during the monitored experiments, enabling continuous acquisition through the same embedded architecture.

Arduino–Raspberry Pi communication was implemented using USB serial communication at 9600 baud. The DO transmitter was accessed separately via Modbus RTU over USB–RS485 at 9600 baud. Dedicated galvanic isolation and quantitative cross-talk testing were not documented.

#### 2.4.1. Fermentation Media, Yeast Strain, and Inoculum Preparation

A commercial active dry yeast strain, *Saccharomyces cerevisiae* var. *bayanus* (PI Bayanus, Chile), was used in the fermentation assays. A synthetic YM medium was employed for the controlled-medium stage and contained 3% malt extract, 3% yeast extract, 5% peptone, and 10% glucose. For the enological assay, Cabernet Sauvignon grape must from Viña Doña Aurora del Peumo, Cauquenes, Maule Region, Chile, was used. The initial grape must contained 264 g/L of D-glucose/D-fructose (GLU–FRU) and 2613 mg/L of primary amino nitrogen (PAN). Ammonium was assessed initially using a Biosystems Y15 automatic analyzer (Biosystems, Barcelona, Spain) and was not detected.

The inoculum was prepared by cultivating PI Bayanus overnight in a 250 mL Erlenmeyer flask containing YM medium at 28 °C and 150 rpm. An appropriate volume of this preculture was subsequently added to each assay to obtain an initial cell concentration of $1\times 10^{6}$ cells/mL.

#### 2.4.2. Fermentation Assay and Complementary Analyses

The fermentation experiment was performed in batch mode with a 20 L working volume and was monitored for six days. Culture samples were collected periodically during this period. Approximately 20% (*w*/*v*) of ammonium sulfate solution was added when nitrogen depletion was detected. These interventions form part of the experimental conditions and should be considered when interpreting short-duration variations in the continuously recorded signals.

Yeast growth was evaluated by optical density at 600 nm (OD_600_) using a Shimadzu UV-1280 UV–Visible spectrophotometer (Shimadzu Corporation, Kyoto, Japan). Samples were centrifuged at 12,000 rpm for 3 min at 10 °C to separate yeast cells from the supernatant, which was retained for chemical analyses. Complementary measurements were performed using a Bante920 pH meter and a Biosystems Y15 automatic analyzer (Biosystems, Spain) to assess acid–base behaviour and GLU–FRU/PAN concentrations, respectively.

These complementary analyses were used to characterize the fermentation experiment; they were not designed as a formal traceable metrological validation of the embedded sensing channels. The resulting experimental configuration enabled the continuous acquisition strategy described in Section 2.5.

The overall experimental procedure, from inoculum preparation to the off-line characterization of the fermentation samples, is summarized in Figure 4.

### 2.5. Data Acquisition and Information Management

Continuous acquisition was designed to preserve the chronological evolution of the monitored variables. Measurements obtained from the sensing subsystem were acquired by the embedded acquisition level and transferred to the processing level, where the information was automatically organized and stored.

Each acquisition sequence incorporated the monitored temperature, pH, DO, and ORP information into the experimental record. Rather than treating the sensing channels as unrelated information sources, the acquisition workflow preserved the temporal order of the variables so that their evolution could be subsequently examined within a common experimental timeline.

The acquired information was stored in structured files organized by acquisition date. This workflow reduced manual transcription and provided the dataset used during the functional evaluation presented in Section 3.

The original Python acquisition routine included a three-second delay between successive cycles; actual intervals also depend on serial communication, sensor response and file-writing overhead. The routine performed basic input checks and organized measurements in date-specific Excel files. Verified channel synchronization, automatic restart recovery, storage-capacity monitoring and drift correction were not evaluated.

### 2.6. Functional Evaluation Strategy

The experimental evaluation was conceived as a functional proof-of-concept assessment of the complete monitoring platform. Its purpose was to determine whether the sensing, acquisition, processing, communication, and information-storage subsystems could operate jointly and continuously under the reported experimental conditions.

The evaluation was performed in three stages consistent with the original experimental procedure: initial verification of the embedded acquisition and communication workflow, operation in the defined synthetic YM medium, and operation under enological conditions using Cabernet Sauvignon grape must. During these stages, the platform recorded temperature, pH, DO, and ORP and generated chronological experimental records.

Accordingly, the evaluation reported here should be interpreted as a laboratory-scale proof-of-concept. Measurement uncertainty, systematic repeatability assessment, long-term drift characterization, comparison against traceable reference instruments, and industrial qualification were not part of the reported experimental protocol.

## 3. Results and Functional Evaluation

The experimental evaluation was conducted to determine whether the sensing, acquisition, processing, and information-management subsystems could operate jointly as a continuous multiparametric monitoring platform. Accordingly, the results are interpreted primarily from a functional perspective and do not constitute a complete metrological characterization of the individual sensing devices.

### 3.1. Embedded Platform Operation and Data Management

During the reported experiments, communication between the acquisition and processing units remained operational, allowing measurements from the different sensing channels to be transferred to the processing unit. The acquired information was automatically organized according to acquisition date and stored for subsequent analysis.

Figure 5 illustrates the implemented information-management workflow. The resulting records preserved the chronological sequence of the experiments and constituted the dataset used for subsequent evaluation.

These observations verify the functional operation of the acquisition chain during the reported tests. Communication reliability, data-loss probability, latency, and timing accuracy were not independently quantified; therefore, the results should be interpreted as evidence of functional continuity rather than quantitative communication performance.

### 3.2. Operation Under Controlled Conditions

Before operation with grape must, preliminary tests were performed using the defined synthetic YM medium (3% malt extract, 3% yeast extract, 5% peptone, and 10% glucose). The purpose of this stage was to verify the coordinated operation of the sensing, acquisition, and processing subsystems in a compositionally defined fermentation medium before the complete platform was used under enological conditions.

The monitored channels generated continuous records during these preliminary tests, and the acquired information was incorporated into the automatic storage workflow. These tests provided functional verification of the complete measurement chain but did not constitute a metrological validation of the individual sensors.

### 3.3. Multiparametric Monitoring During Wine Fermentation

Following the preliminary tests, the complete platform was operated with Cabernet Sauvignon grape must under enological conditions to evaluate its ability to continuously record changes in the physicochemical environment during wine fermentation. The batch assay used a 20 L working volume, an initial inoculum of $1\times 10^{6}$ cells/mL of *S. cerevisiae* var. *bayanus*, and a six-day monitoring period. Figure 6 shows the temporal evolution of temperature, pH, oxidation–reduction potential, and dissolved oxygen.

The four variables exhibited different temporal patterns. The following analysis distinguishes between trends directly observable in the experimental records and their possible physicochemical interpretation. The latter is discussed in relation to the enological literature without assuming that every variation observed in the sensor signals necessarily originated from the fermentation process.

#### 3.3.1. Temperature Evolution

As shown in Figure 6a, temperature varied throughout the monitored period. The profile contains an initially higher temperature followed by a general decrease with shorter-term variations occurring during the experiment. Temperature influences yeast metabolism, fermentation kinetics, and wine composition [1,2]. In the present study, however, no independent thermal-balance analysis was performed; therefore, the relative contributions of microbial activity, ambient conditions, and other experimental factors cannot be separated from the available data.

From a functional perspective, the relevant observation is that the temperature channel remained operational and preserved the temporal evolution of the thermal conditions throughout the monitoring period.

#### 3.3.2. pH Evolution

The pH profile in Figure 6b exhibited a progressive decrease during the initial part of the experiment, which was followed by comparatively smaller variations. However, the absolute values recorded during the initial stage, including values above the range typically expected for grape must, must be interpreted with caution. Since synchronized measurements against traceable reference instrumentation were not performed, the available experimental data do not allow determining whether these values reflected the actual medium conditions, sensor calibration effects, measurement drift, or other instrumental factors. Consequently, the pH record is considered here as evidence of continuous signal acquisition and temporal tracking rather than as a validated quantitative measurement of must pH [3,4,5].

The unusually high initial pH readings may reflect calibration offset, electrode stabilization following immersion, or analog signal-conditioning effects. Since these factors were not independently characterized, the initial absolute values should not be interpreted as validated must pH.

The continuous profile demonstrates the capability of the platform to follow gradual changes in this variable. However, no parallel measurement against traceable reference instrumentation was performed; therefore, the observed trend supports functional monitoring capability but does not establish the analytical accuracy of the pH sensing channel.

#### 3.3.3. Oxidation–Reduction Potential Evolution

The ORP record in Figure 6c exhibits a more complex temporal behavior. The overall profile evolves toward negative potential values, which is consistent with a transition toward more reducing conditions in the monitored medium [9,10,11,12].

Several short-duration excursions toward more positive values are superimposed on this general behavior. ORP can be influenced by oxygen availability, chemical composition, mixing, and microbial activity. The experimental configuration does not allow the origin of each transient excursion to be uniquely identified. They may represent actual short-term changes in the medium, operational disturbances, local conditions near the sensing element, or measurement-related effects. Although complementary off-line analyses were available for fermentation characterization, no event-synchronized reference measurement or operational record was available for these ORP excursions; assigning a particular mechanism to them would therefore be speculative.

The ORP result therefore demonstrates that the platform preserved both the longer-term signal evolution and shorter-duration variations. Determining the physicochemical significance of individual transients requires additional controlled experiments and reference measurements.

#### 3.3.4. Dissolved Oxygen Evolution

The DO profile in Figure 6d remains near the lower part of its recorded range during a substantial portion of the experiment, while several transient increases are also evident. The predominance of low DO values is qualitatively compatible with decreasing oxygen availability as alcoholic fermentation proceeds. Oxygen availability influences yeast metabolism and wine properties, and even relatively small oxygen inputs during winery operations can modify the oxygen exposure of the product [6,7]. Recent instrumented-tank studies also demonstrate the value of following oxygen and carbon-dioxide evolution in real time during vinification [8].

As observed for ORP, the transient increases require cautious interpretation. The available experimental information does not establish whether these excursions resulted from temporary oxygen incorporation, manipulation or mixing, local conditions around the probe, or measurement-related effects. Their specific origin cannot therefore be assigned from the present dataset.

Their detection nevertheless illustrates an important characteristic of continuous acquisition: short-duration events are preserved in the temporal record and could be missed by widely separated discrete measurements. This observation does not establish the biological significance of the events but demonstrates the temporal information retained by the monitoring workflow. In addition, changes in the ORP and DO profiles were observed upon addition of the 20% ammonium sulfate solution, and no other manual operation was performed during that addition. This reported temporal association does not establish a biochemical cause: local mixing, changes in medium composition, oxygen incorporation and sensor-related artifacts cannot be excluded without synchronized event logs and reference measurements.

#### 3.3.5. Combined Interpretation of the Monitored Variables

Taken together, the four profiles demonstrate the simultaneous and continuous acquisition of complementary physicochemical information during wine fermentation. Temperature and pH show comparatively gradual temporal variations, whereas ORP and DO contain both longer-term trends and shorter-duration fluctuations. These differences reinforce the rationale for multiparametric monitoring because the physicochemical evolution of the fermentation environment cannot be represented by a single measured variable. Comparable studies in wine and other fruit-wine fermentations have likewise combined multiple physicochemical or spectroscopic descriptors to follow process evolution, supporting the value of temporally resolved multivariable information [5,17].

The experimental profiles demonstrate that the platform can acquire and preserve multiparametric temporal information. They do not demonstrate that every recorded variation originates from a specific biological or chemical fermentation mechanism.

The present platform primarily provides exploratory chronological records that may help identify intervals for targeted sampling. Independently measured sugar consumption, assimilable nitrogen and yeast-cell concentration may contextualize the fermentation trajectory, but they cannot by themselves validate individual ORP or DO transients or establish their causal origin.

### 3.4. Integrated Functional Assessment

The results obtained during the different experimental stages provide a consistent basis for evaluating the functional operation of the proposed platform. During the reported tests, sensing, acquisition, processing, and information-management functions operated jointly and generated chronological records of the monitored variables.

At the architectural level, measurements acquired from the sensing subsystem were transferred to the processing unit and automatically stored. At the experimental level, the same architecture continuously recorded temperature, pH, ORP, and DO during the fermentation experiment. The principal outcome is therefore the demonstration of functional integration rather than metrological superiority.

The experiments show that commercially available sensing devices can be integrated with a distributed embedded acquisition and processing architecture to generate structured multiparametric records during laboratory-scale wine fermentation. The present results demonstrate functional feasibility at the proof-of-concept level; they do not establish analytical accuracy, precision, repeatability, measurement uncertainty, long-term sensor stability, or industrial qualification.

### 3.5. Scope and Limitations

The experimental evidence should be interpreted within the scope of a laboratory-scale proof of concept. First, the experiments were conducted in a laboratory reactor with an approximate nominal capacity of 25 L and a 20 L working volume. Although this configuration allowed operation under enological conditions, the results cannot be directly extrapolated to industrial fermentation tanks, where thermal, hydrodynamic, and operational conditions differ.

Second, the sensing subsystem was not subjected to complete metrological characterization. The study did not include systematic uncertainty estimation, repeatability assessment, long-term drift characterization, or quantitative comparison against traceable reference instrumentation. This limitation is particularly relevant when interpreting the ORP and DO transients observed during fermentation.

Third, continuous exposure to the fermentation medium may affect sensor response over prolonged operation. The experiments reported here were not designed to quantify this effect. Long-term studies including systematic calibration and drift assessment are therefore required before routine quantitative monitoring can be claimed.

Additional engineering development would also be required for deployment outside the laboratory. The electronic components require appropriate encapsulation and environmental protection for routine operation under winery conditions. The present implementation is primarily oriented toward data acquisition and storage and does not include an integrated graphical interface for the interactive visualization of all monitored variables.

Finally, the platform was developed for monitoring rather than closed-loop process control. No automatic control actions based on the acquired variables were evaluated. Likewise, although the generated datasets may support future predictive or data-driven analyses, artificial intelligence, predictive modeling, and automatic supervisory functions were not implemented and therefore do not constitute demonstrated capabilities of the present prototype.

Future work should include sensor calibration and drift characterization, quality flags for missing or repeated data, communication-health monitoring, synchronized intervention logs, and periodic reference sampling. Industrial transfer would additionally require sensor-placement studies, fouling assessment, cleaning protocols, environmental protection and independent safety measures before any closed-loop control.

Taken together, these considerations define the present technological maturity of the system. The results support its use as a laboratory-scale infrastructure for continuous multiparametric monitoring, while metrological characterization, long-term stability assessment, industrial-scale testing, environmental protection, and interface development remain necessary steps toward a more mature implementation.

The modular acquisition architecture may be adapted to other fermentation or bioprocess environments; however, compatibility with different media, viscosity, fouling behavior and sensor requirements has not been demonstrated. The estimated cost of developing the laboratory-scale prototype was CLP 536,090, which should not be interpreted as a complete industrial cost or evidence of economic superiority.

## 4. Conclusions

This work presented the design and functional evaluation of an embedded multiparametric monitoring platform for the continuous observation of physicochemical variables during laboratory-scale fermentation experiments. The architecture integrates sensing, embedded acquisition, local processing, and automatic information management within a single experimental infrastructure capable of recording temperature, pH, ORP, and DO throughout the monitored process.

The experimental results demonstrate the functional feasibility of the proposed approach at the proof-of-concept level. During the reported tests, the acquisition and processing units operated jointly, the monitored variables were continuously recorded, and the resulting information was chronologically organized and stored for subsequent analysis. Under enological conditions, the platform captured distinct temporal patterns, including gradual changes in temperature and pH and shorter-duration variations in ORP and DO.

The principal contribution therefore lies in the functional integration of commercially available sensing technologies within a distributed embedded architecture for continuous multiparametric monitoring. The developed platform should be regarded as an experimental monitoring infrastructure rather than a fully validated industrial instrument. Its present contribution is associated with integrating sensing, acquisition, processing, and data organization into a unified workflow that generates structured experimental records.

The results also highlight the importance of distinguishing continuous signal acquisition from metrological validation. The experiments demonstrate that the platform can detect and preserve temporal variations in the monitored variables; however, they do not establish the analytical accuracy, precision, repeatability, measurement uncertainty, or long-term stability of the individual sensors. In particular, the transient variations observed in ORP and DO could not be uniquely associated with specific biological, physicochemical, or measurement-related phenomena from the available information.

Accordingly, the present prototype represents a laboratory-scale functional proof-of-concept and should not yet be considered an industrially validated monitoring solution. Further development should focus on quantitative sensor characterization against traceable reference instrumentation, long-term drift and repeatability assessment, evaluation under a broader range of fermentation conditions, environmental protection of the electronic components, and subsequent testing at larger production scales.

Within these boundaries, the results support the feasibility of using a distributed embedded architecture as the basis for continuous multiparametric monitoring in experimental enology. The platform provides a structured technological foundation for subsequent developments in fermentation supervision, quantitative process characterization, and, after appropriate validation, future data-driven monitoring strategies.

## Figures and Tables

**Figure 1:**
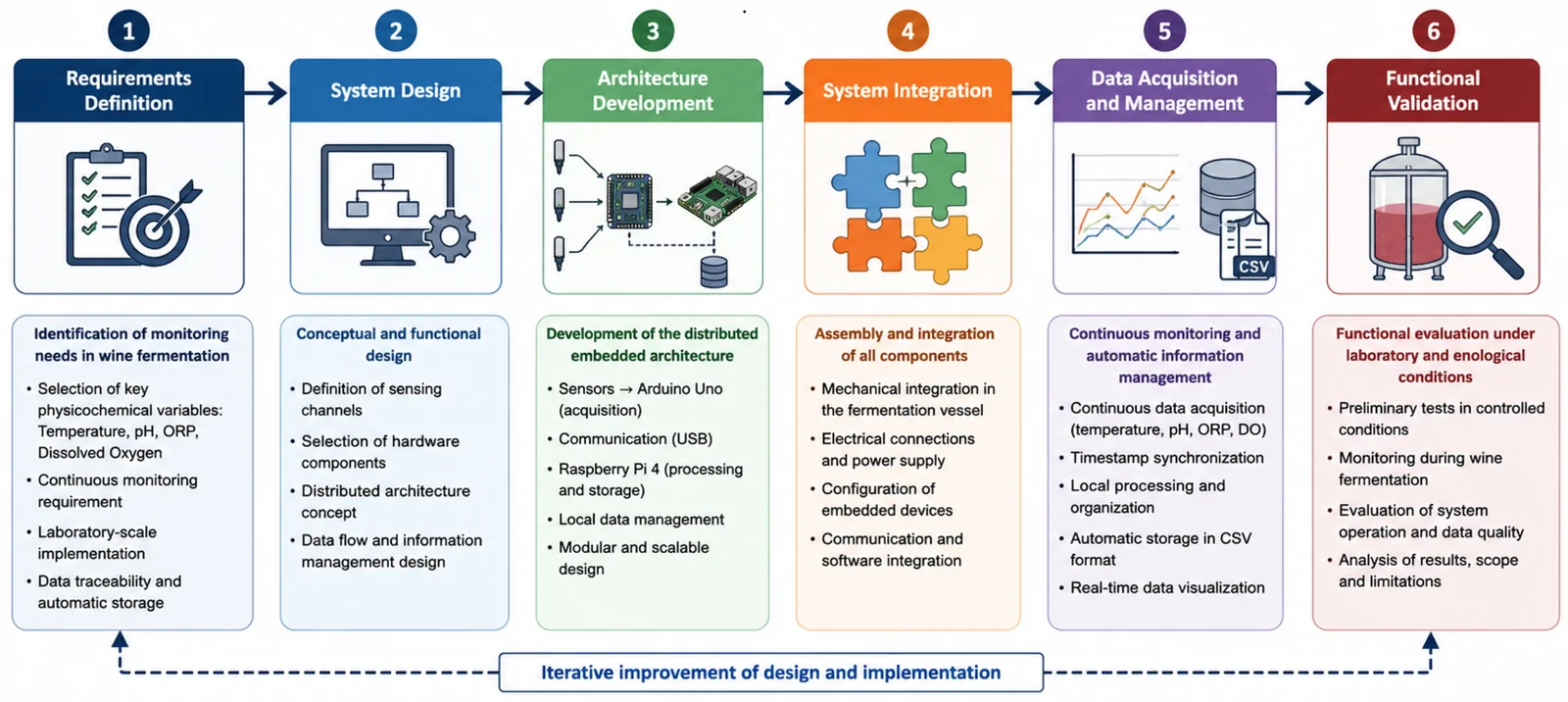
Methodological workflow adopted for the design, implementation, and functional evaluation of the embedded multiparametric monitoring platform. The workflow integrates requirements definition, system design, distributed embedded architecture development, system integration, data acquisition and information management, and functional evaluation under controlled and enological conditions.

**Figure 2:**
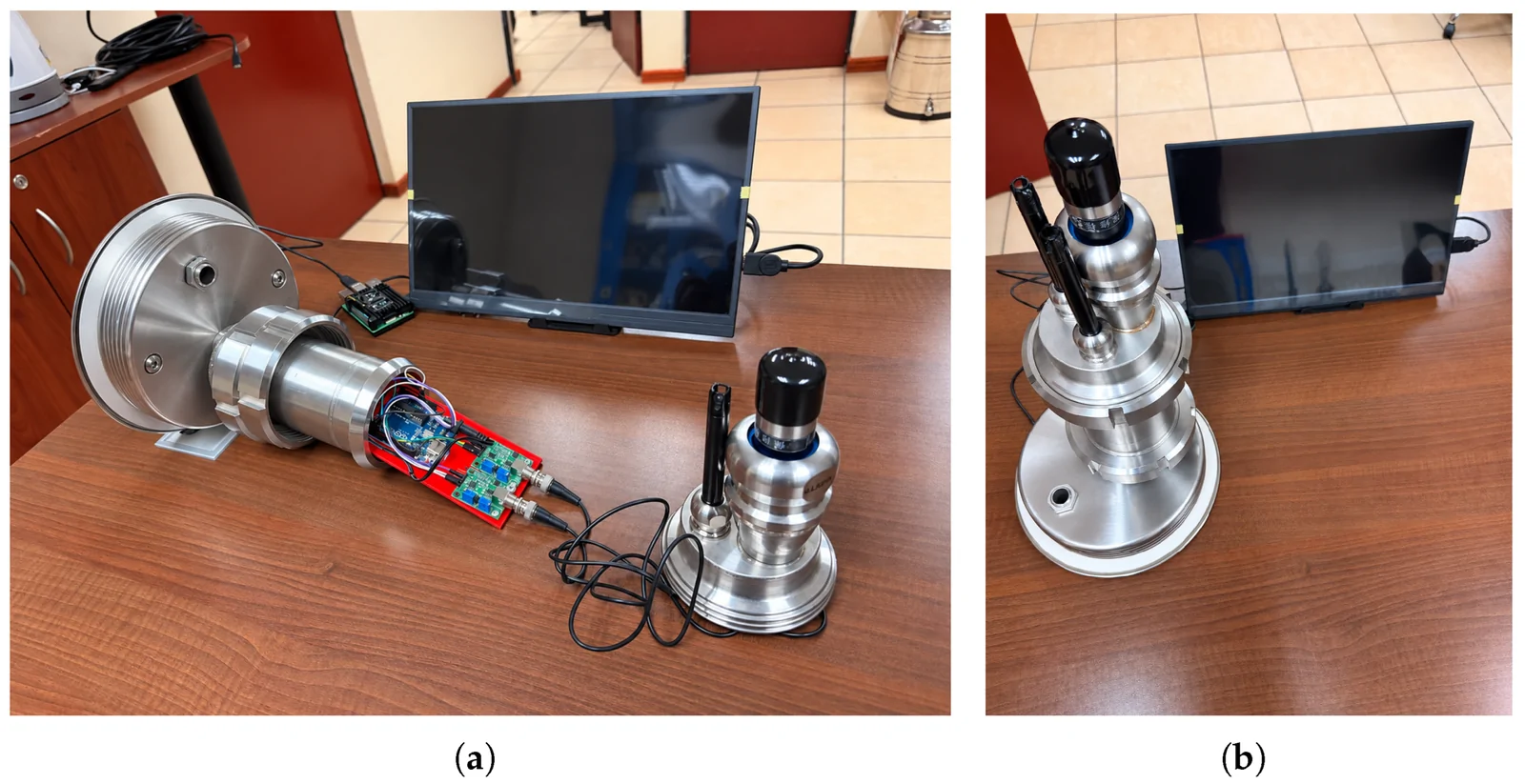
Experimental prototype of the wine monitoring system. (**a**) Disassembled configuration showing the stainless-steel sensing module, electronic interface, and data acquisition components. (**b**) Assembled configuration of the monitoring module, illustrating the final integration of the sensing and electronic components for in situ measurements during the winemaking process.

**Figure 3:**
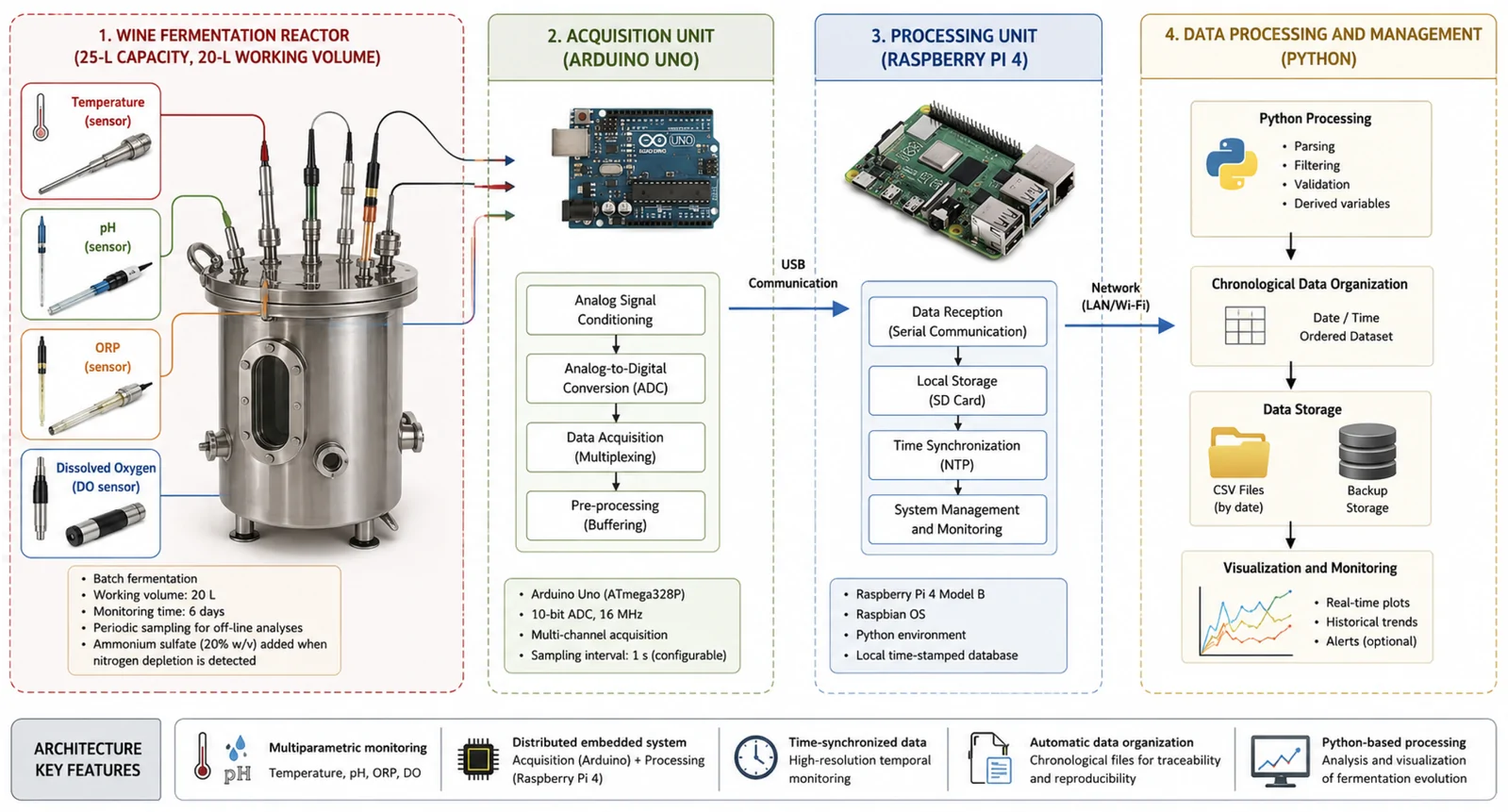
Distributed embedded monitoring architecture for wine fermentation. The multiparametric sensing system integrates temperature, pH, oxidation–reduction potential (ORP), and dissolved oxygen (DO) measurements within the stainless-steel fermentation reactor. Sensor signals are acquired by an Arduino Uno, which operates as the acquisition unit and provides the interface between the sensing channels and the processing subsystem. The acquired data are subsequently transferred to a Raspberry Pi 4, which performs local processing, management, and storage. Python-based routines are used for data processing and chronological organization of the measurements, generating structured records for subsequent analysis. This distributed architecture separates sensor acquisition from data processing and management, providing continuous multiparametric monitoring and automatic storage of the fermentation data.

**Figure 4:**
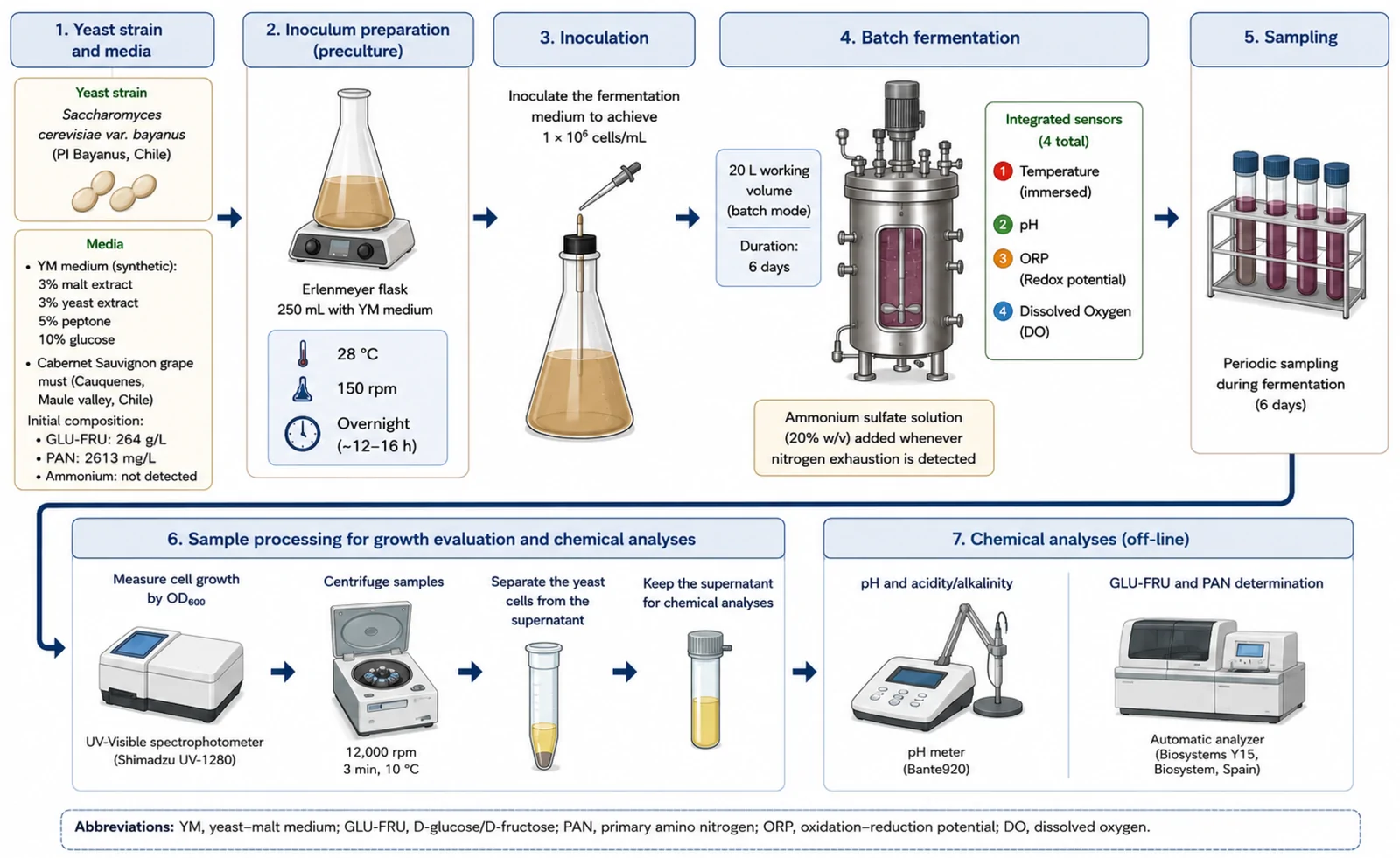
Schematic representation of the experimental protocol used for the fermentation assays. The workflow includes the selection of the yeast strain and fermentation media, inoculum preparation, inoculation, batch fermentation, periodic sampling, and subsequent off-line analyses. A commercial *Saccharomyces cerevisiae* var. *bayanus* strain (PI Bayanus, Chile) was precultured in YM medium at 28 °C and 150 rpm overnight and subsequently inoculated to achieve an initial concentration of $1\times 10^{6}$ cells/mL. Fermentation was performed in batch mode using a 20 L working volume and monitored for six days. Periodically collected samples were used to determine yeast growth by optical density at 600 nm (OD_600_) and were centrifuged to obtain the supernatant for chemical analyses. Off-line measurements included pH and acidity/alkalinity determination using a Bante920 pH meter and GLU-FRU and primary amino nitrogen (PAN) determination using a Biosystems Y15 automatic analyzer. A 20% (*w*/*v*) ammonium sulfate solution was added when nitrogen depletion was detected.

**Figure 5:**
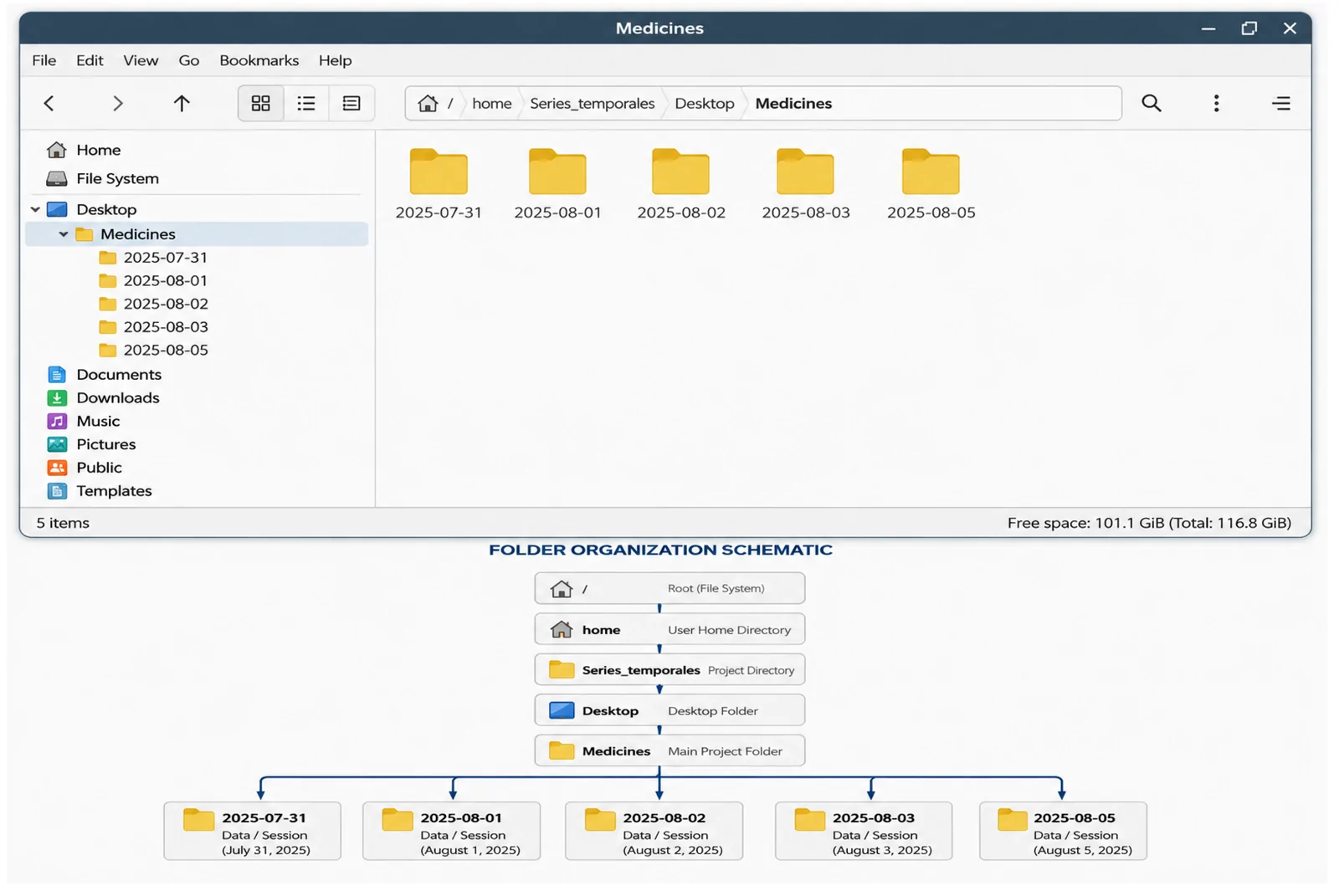
Information-management workflow implemented in the monitoring platform. Measurements acquired from the sensing subsystem are transferred to the processing unit and chronologically organized for automatic storage and subsequent experimental analysis.

**Figure 6:**
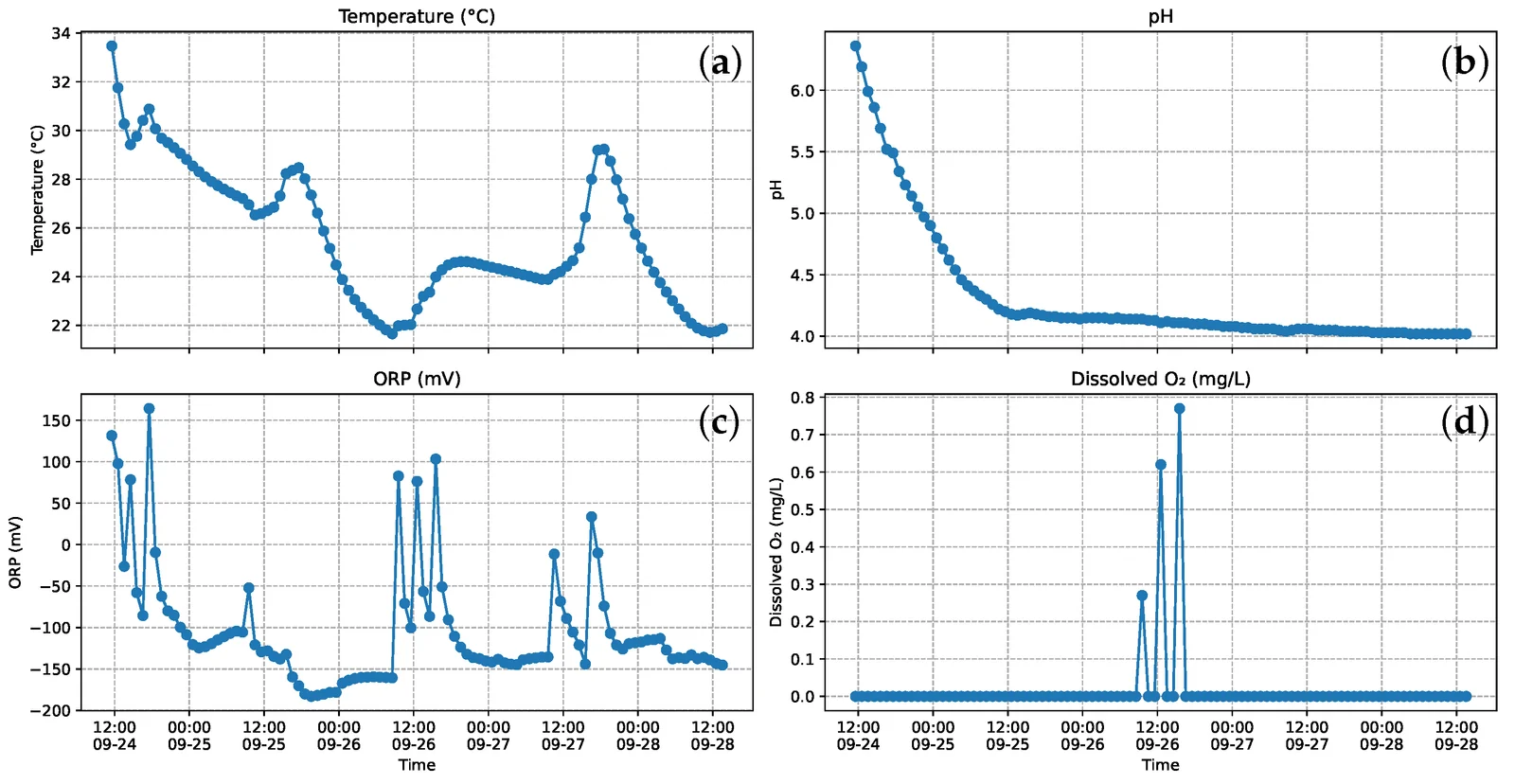
Temporal evolution of the physicochemical variables recorded during the wine fermentation experiment: (**a**) temperature, (**b**) pH, (**c**) oxidation-reduction potential (ORP), and (**d**) dissolved oxygen (DO). The profiles represent the continuous multiparametric information generated by the proposed monitoring platform. Temperature and pH exhibit comparatively gradual temporal changes, whereas the ORP and DO records also contain short-duration variations whose origin was not independently established in the present study.

**Table 1:** Main components of the embedded multiparametric monitoring platform. Unavailable model or manufacturer information is explicitly indicated.

Variable/Function	Component	Model	Manufacturer
Temperature	Digital temperature sensor	DS18B20	Maxim Integrated (San Jose, CA, USA)/Analog Devices (Wilmington, MA, USA)
pH	pH electrode + BNC interface module	Not reported	Not reported
ORP	ORP electrode/sensing module	Not reported	Not reported
Dissolved oxygen	Fluorescence DO transmitter	RS-LDO-N01-1	Renke Control Technology (Jinan, China)
Acquisition	Arduino Uno	Uno	Arduino (Chiasso, Switzerland)
Processing	Raspberry Pi 4	Raspberry Pi 4	Raspberry Pi (Cambridge, UK)

## Data Availability

The data presented in this study are available on request from the corresponding author.
